# Molybdate transporter ModABC is important for *Pseudomonas aeruginosa* chronic lung infection

**DOI:** 10.1186/s13104-016-1840-x

**Published:** 2016-01-12

**Authors:** Simone Périnet, Julie Jeukens, Irena Kukavica-Ibrulj, Myriam M. Ouellet, Steve J. Charette, Roger C. Levesque

**Affiliations:** Institut de Biologie Intégrative et des Systèmes, Université Laval, Québec, QC Canada; Département de microbiologie-infectiologie-immunologie, Faculté de Médecine, Université Laval, Québec, QC Canada; Département de biochimie, de microbiologie et de bio-informatique, Faculté des Sciences et de Génie, Université Laval, Québec, QC Canada; Centre de recherche de l’Institut universitaire de cardiologie et de pneumologie de Québec (CRIUCPQ), Quebec, QC Canada

**Keywords:** ModA, Molybdate, *Pseudomonas aeruginosa*, Cystic fibrosis, Anaerobic conditions, Biofilm, Chronic lung infection, Animal model

## Abstract

**Background:**

Mechanisms underlying the success of *Pseudomonas aeruginosa* in chronic lung infection among cystic fibrosis (CF) patients are poorly defined. The *modA* gene was previously linked to in vivo competitiveness of *P. aeruginosa* by a genetic screening in the rat lung. This gene encodes a subunit of transporter ModABC, which is responsible for extracellular uptake of molybdate. This compound is essential for molybdoenzymes, including nitrate reductases. Since anaerobic growth conditions are known to occur during CF chronic lung infection, inactivation of a molybdate transporter could inhibit proliferation through the inactivation of denitrification enzymes. Hence, we performed phenotypic characterization of a *modA* mutant strain obtained by signature-tagged mutagenesis (STM_*modA*) and assessed its virulence in vivo with two host models.

**Results:**

The STM_*modA* mutant was in fact defective for anaerobic growth and unable to use nitrates in the growth medium for anaerobic respiration. Bacterial growth and nitrate usage were restored when the medium was supplemented with molybdate. Most significantly, the mutant strain showed reduced virulence compared to wild-type strain PAO1 according to a competitive index in the rat model of chronic lung infection and a predation assay with *Dictyostelium discoideum* amoebae. As the latter took place in aerobic conditions, the in vivo impact of the mutation in *modA* appears to extend beyond its effect on anaerobic growth.

**Conclusions:**

These results support the *modABC*-encoded transporter as important for the pathogenesis of *P. aeruginosa*, and suggest that enzymatic machinery implicated in anaerobic growth during chronic lung infection in CF merits further investigation as a potential target for therapeutic intervention.

**Electronic supplementary material:**

The online version of this article (doi:10.1186/s13104-016-1840-x) contains supplementary material, which is available to authorized users.

## Background

*Pseudomonas aeruginosa* is an environmental bacterium and the most common cause of chronic lung infection among cystic fibrosis (CF) patients [1]. Its success in causing opportunistic infection and its persistence capacities are presumably attributable to a large 6.3–6.9 Mbp genome regulated by more than 550 transcriptional regulators, which allow adaptation to diverse environments and growth conditions [2, 3]. *P. aeruginosa* also produces a wide repertoire of molecules and sensors for nutrient uptake, adhesion, mobility, biofilm formation and antibiotic resistance that are all key components of in vivo proliferation [4].

It was shown that the CF mucus is oxygen-depleted [5] and may carry strict anaerobes [6]. Hence, during chronic lung infection in CF, *P. aeruginosa* is exposed to microaerophilic [7] or anaerobic conditions [8], which are well suited for biofilm formation [9]. Denitrification, the main source of energy production under anaerobic conditions, is based upon the reduction of oxidized forms of nitrogen (preferentially nitrates, NO_3_) by metalloenzymes such as nitrate reductase [10, 11]. Molybdate (MoO_4_^2−^) is the usable form of trace-element molybdenum (Mo) [12] and resembles sulfate, phosphate, tungstate and vanadate in molecular size, shape, charge and hydrogen-binding properties [13]. Mo is incorporated into the molybdenum cofactor (MoCo) and was found to be essential for the activity of molybdoenzymes. These enzymes catalyze various oxidation/reduction reactions and are implicated in the metabolism of nitrogen, carbon and sulfur. All nitrate reductases required for *P.aeruginosa* anaerobic growth require a MoCo cofactor [11], which can contain either Mo or tungsten (W) [14].

A previous PCR-based signature-tagged mutagenesis (PCR-STM) mutant screen allowed the identification of 148 genes presumably essential for in vivo survival of *P. aeruginosa* PAO1 in a rat model of chronic lung infection [15]. One of these genes was *modA*. In *E.* *coli*, molybdate and tungstate are internalized using an ATP-binding cassette transporter, ModABC [16], where ModA is the periplasmic binding protein with a high affinity for Mo/W, ModB is the integral membrane channel protein and ModC is the energizer protein [17]. In *P. aeruginosa*, *modA* was recently demonstrated to be essential for molybdate acquisition and anaerobic growth using a deletion mutant [18]. Here, with the goal of gaining further information on the role of ModABC in the virulence of *P. aeruginosa,* we present a characterization of the PAO1 transposon mutant STM_*modA.* We then assess the relevance of *modA* as a therapeutic target by testing virulence attenuation of STM_*modA* in two host models: the rat model of chronic lung infection, which best represents the context of CF lung infections, and the amoeba predation assay, which takes place in aerobic conditions. Results suggest that *modA* is important for the pathogenesis of *P. aeruginosa* in both host models.

## Methods

### Bacterial strains, plasmids, primers and culture conditions

Bacterial strains and plasmids used in this study are listed in Table 1. The STM_*modA* mutant was constructed and identified in the context of PCR-based STM using strain PAO1 [15, 19, 20], a laboratory strain previously shown to be appropriate and comparable to a CF isolate (LESB58) for the study of virulence using the rat model of chronic lung infection [21]. Briefly, a tagged-miniTn*5*Km2 suicide plasmid was transferred into strain PAO1 by conjugation, generating thousands of mutants, each containing a unique random mutation. Mutant strains were then negatively screened in pools of 72 in the rat model of chronic lung infection to identify defective mutants for in vivo maintenance via multiplex PCR. Mutation of *modA* in the STM_*modA* mutant strain was confirmed by cloning using a plasmid rescue based on the miniTn*5*Km2 antibiotic resistance marker followed by sequencing and alignment on the PAO1 genome available at the *Pseudomonas* genome database [22].

**Table 1:** Strains and plasmids

Strain or plasmid	Relevant characteristics	Source or reference
*E. coli* strain		
NEB 5α	Chemically competent cells	New England Biolabs
*P. aeruginosa* strains		
PAO1	PAO1293, Cm^S^, wild-type, derivative of PAO2 which originates from PAO1	[48]
STM_*modA*	PAO1 STM_*modA*::miniTn*5*-Km2 mutant inactivating *modA* (PA1863), Km^R^	[15]
Complemented STM_*modA*	PAO1 STM_*modA* with pUCP19::*modABC* plasmid (PA1863, PA1862, PA1861)	This study
Plasmids		
pUCP19	Cb^R^, cloning vector and used for CI	[49]
pUCP19 ::*modABC*	Cb^R^, Km^R^, *modABC* genes on pUCP19 vector	This study

For routine cultures, *P. aeruginosa* and *E.coli* were grown aerobically at 37 °C in tryptic soy broth (TSB) or Luria–Bertani (LB) broth (EMD Serono). When needed, culture medium was supplemented with 1.5 (w/v) % bacto agar, kanamycin (Km 150 μg ml^−1^ for STM_*modA*; Calbiochem), ampicillin (Ap 100 μg ml^−1^ for DH10B transformants; Sigma-Aldrich) or carbenicillin (Cb 200 μg ml^−1^ for *P.aeruginosa* transformants; Thermo-Fisher).

For growth experiments, *P. aeruginosa* was grown aerobically under vigorous shaking (250 rpm) in 100 ml LB using a 1:10 pre-culture to Erlenmeyer volume ratio. For anaerobic growth, nephelo flasks sealed with silicone stoppers containing 100 ml LB were flushed with argon for 45 min and needle-inoculated with 1 ml of pre-culture before incubation with weak agitation (120 rpm) to prevent precipitation. Media for aerobic and anaerobic growth were supplemented with potassium nitrate (KNO_3_; 15 mM; Merck) and, when needed, sodium molybdate (100 μM; Sigma-Aldrich). Growth was monitored by spectrophotometric measurements of optical density at 600 nm. Growth experiments were repeated three times.

Restriction enzymes, Q5 polymerase and Gibson assembly cloning kit were purchased from New England Biolabs. The QIAprep Spin Miniprep Kit (Qiagen) was used for plasmid isolation and the DNeasy Blood and Tissue kit (Qiagen) was used for genomic DNA isolation. PCR reactions were performed in an iCycler (Bio-Rad); primers used in this study are listed in Additional file 1.

### Spectrophotometric determination of nitrate in culture medium after anaerobic growth

To determine if the STM_*modA* mutant was able to use nitrates during anaerobic growth, the concentration of residual nitrates in the medium was quantified after overnight growth based on the principle of chemical reduction of nitrate and its spectrophotometric detection using the Griess reaction [23]. Vanadium (III) chloride (Sigma-Aldrich) was used for the reduction of nitrates to nitrites. Sulfanilamide and N-(1-naphtyl)-ethylenediamine (Sigma-Aldrich) were used in the composition of the Griess reagent. Nitrite concentration was subtracted from the total nitrate and nitrite concentration to obtain the nitrate concentration alone.

### Complementation of the STM_*modA* mutation

Cloning of the *modABC* operon into the pUCP19 vector was done using the Gibson Assembly Master Mix (New England Biolabs) following manufacturer’s instructions. Two overlapping PCR fragments (Additional file 1) containing the entire *modABC* operon plus a 448 nt upstream region (2.1 kb final insert size) and an overlapping section of the *Kpn*I and *Pst*I restriction sites were ligated with the digested vector. The recombinant plasmid was introduced by heat shock into *E.* *coli* NEB 5α chemically competent cells (Table 1). The plasmid transfer was first confirmed by *Eco*RI and *Pst*I digestion followed by DNA sequencing. The plasmid was then recovered and electroporated into the *P.aeruginosa* STM_*modA* mutant. Plasmid insertion in the mutant strain was confirmed using digestions with the same restriction enzymes.

### Biofilm formation assay

Multiple phenotypic tests were performed for this study (see reference [24] for more information on the protocols used), but only biofilm formation showed variation among strains. To measure the quantity of biofilm produced by wild-type strain PAO1, STM_*modA*, and the complemented STM_*modA*, a 96-plate rapid biofilm formation assay was performed as previously described [25]. Briefly, strains were grown overnight in LB supplemented when needed with Km or Cb. The M63 culture medium for biofilm formation [25] was supplemented when needed with 100 μM sodium molybdate and 15 mM KNO_3_. Biofilms were stained with crystal violet after a 6-hour static incubation time. Biofilm formation was quantified after stain dissolution in 2 × 200 μl 95 % v/v ethanol [26]. The experiment was done three times, with eight repetitions for each strain and condition. Statistical significance was assessed using an ANOVA in GraphPad Prism 6.0.

## Ethics statement

The rat model in this study was used in a protocol approved by the “Comité de protection des animaux de l’Université Laval” (certificate 2011194-3, IACUC is a Canadian Council on Animal Care certificate holder).

### Preparation of agarose bead-embedded bacteria and in vivo competitive index

The previously described rat model of chronic lung infection [27] using agarose bead-embedded bacteria was optimized as described elsewhere [20]. Sprague–Dawley rats of 450–500 g in weight were sedated (ketamine/xylazine IP injection, 10 mg/100 g) and a local anesthetic (lidocaine) was applied to the vocal cords. Animals were then intubated and inoculated with 120 μl of bead preparation containing PAO1 + pUCP19 (Cb^R^) strain and the STM_*modA* mutant in equal parts (1.6 × 10^6^ CFU ml^−1^ per strain) for competitive index (CI) determination. At day 7 post-infection, rats were euthanized by barbiturate overdose (Euthanyl IP injection, 120 mg/kg) and homogenized lungs were diluted and plated in triplicate on TSA to quantify the total number of viable *P.aeruginosa* cells (TSA supplemented with 200 μg Cb ml^−1^ for the wild-type selection or TSA supplemented with 150 μg Km ml^−1^ for the mutant selection). In vivo CI was calculated as the ratio of mutant to wild-type bacteria recovered in vivo and adjusted according to the input ratio. The final CI data was represented as the geometric mean for each group of six animals and statistical significance was assessed with a two-tailed Mann–Whitney test on GraphPad Prism 6.0 software.

### Amoeba predation assay

A predation assay was used to determine the bacterial capacity to resist to amoeba grazing [28]. The *D. discoideum* amoeba (DH1-10) was grown in HL5 medium (14.3 g l^−1^ of bactopeptone, 7.15 g l^−1^ of yeast extract, 18 g l^−1^ of d-(+)-monohydrate maltose, 0.641 g l^−1^ of Na_2_HPO_4_•2H_2_O, and 0.490 g l^−1^ of KH_2_PO_4_) [29] supplemented with 15 µg ml^−1^ tetracycline. The confluence of amoebae was about 60 % the day of the experiment. A volume of 300 µL of *P.aeruginosa* liquid pre-culture (OD_600_ = 0.9) was spread in a uniform lawn on a Petri dish containing SM 1/10 agar (1 g l^−1^ of bactopeptone, 0.1 g l^−1^ of yeast extract, 0.22 g l^−1^ of KH_2_PO_4_, 0.1 g l^−1^ of K_2_HPO_4_, 0.1 g l^−1^ of MgSO_4_∙7H_2_O, 2 g l^−1^ of Bacto agar, and 1 g l^−1^ of glucose). Serial 1/10 dilutions of 50,000 amoeba cells/5 µL down to 0 cell/5 µl were deposited on the bacterial lawn. Petri dishes were incubated at 21 °C for 7 days and phagocytic plaques due to amoeba grazing were monitored. The experiment was repeated three times.

## Results and discussion

### Genetic characterization of the *P.aeruginosa* STM_*modA* mutant

**Fig. 1:**
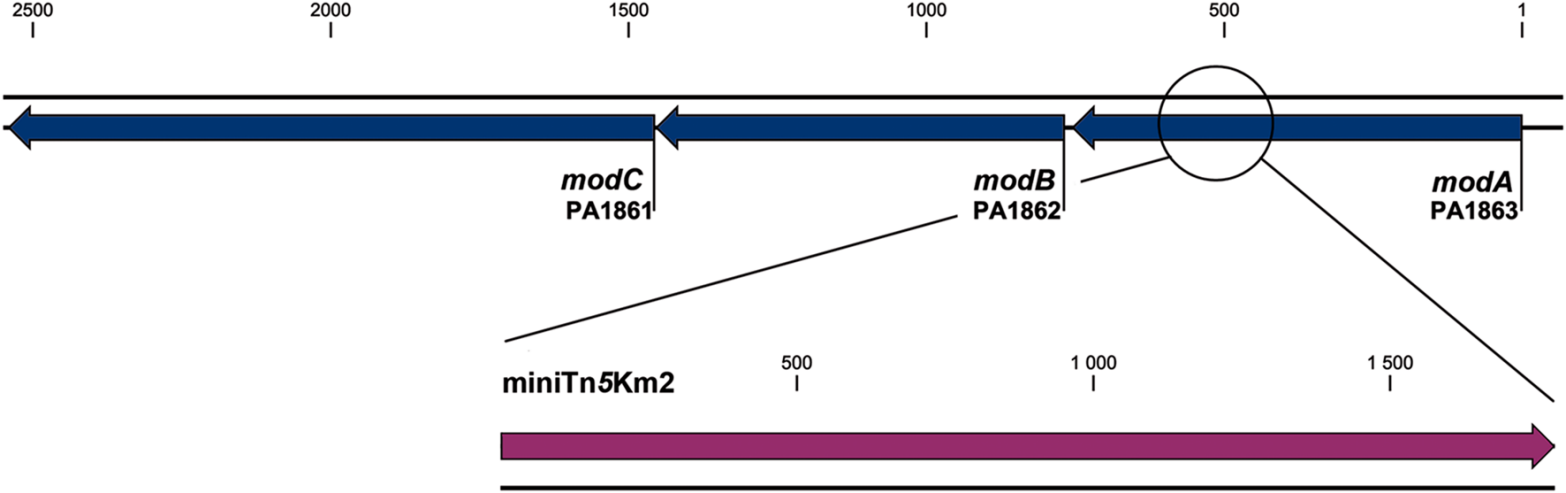
Genomic organization of the *P. aeruginosa* *modABC* operon in the STM_*modA* mutant strain. The miniTn*5* transposon is inserted at position 540. The *modA* gene encodes a 252 amino acid molybdate-binding periplasmic protein precursor. Products of *modB* and *modC* are the molybdenum transport protein ModB and ModC, respectively. *Arrows* indicate the direction of transcription and numbers are relative to the transcription start site of *modA*

Sequencing of the mutated *modA* gene showed that the miniTn*5* insertion and its typical 9-pb flanking duplication [30] were inserted in the open reading frame (ORF) of *modA* at position 540 (Fig. 1), and introduced a stop codon assumed to prematurely interrupt translation of the protein. In the 6.3 Mbp genome of *P.aeruginosa* strain PAO1*, modA* (PA1863) is encoded in a putative operon with *modB* (PA1862) and *modC* (PA1861) (Fig. 1). Their products correspond to the 26.4 kDa (252 AA) molybdate-binding periplasmic protein precursor ModA, molybdenum membrane transport protein ModB (24.4 kDa) and ATPase ModC (39.8 kDa). These annotations are based on AA identity with ModABC from *E.coli* [31], where the *modABC* transcription unit codes for an ATP-binding cassette (ABC) transporter of molybdate, and were confirmed by Pederick and colleagues [18] using a *modA* deletion mutant. Since ABC transporters require a binding protein to present the substrate to the cargo membrane protein(s) [32], ModABC is expected to be non-functional in the STM_*modA* mutant.

### *modA* is required for anaerobic growth and nitrate utilization

**Fig. 2:**
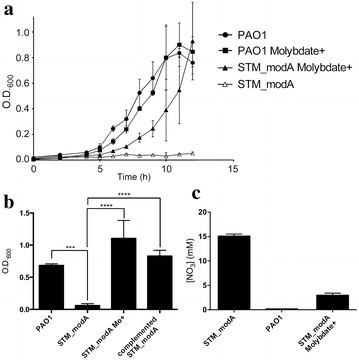
STM_*modA* is unable to perform anaerobic growth and use nitrates without molybdate supplementation. **a** anaerobic growth curves in LB medium supplemented with 15 mM KNO_3_, plus 100 μM sodium molybdate when indicated. STM_*modA* is unable to grow in anaerobic conditions with nitrates as the terminal electron acceptor without molybdate supplementation. Results are from three independent experiments; error bars represent standard deviation **b** anaerobic overnight growth in LB medium supplemented with 15 mM KNO_3_, plus 100 μM sodium molybdate when indicated. Addition of molybdate restored growth in the STM_*modA* mutant. The complemented STM_*modA* strain showed growth without molybdate supplementation. Statistical significance was assessed using an ANOVA in GraphPad prism 6.0 (*** p = 0.0003; **** p < 0.0001). **c** Residual nitrate dosage in the medium after overnight growth using the Griess reaction. Nitrates levels are inversely proportional to nitrate uptake and utilization. Adding molybdate to the medium restored the STM_*modA* mutant’s nitrate consumption. Results for **b** and **c** are for three technical replicates in one of three consistent experiments

Under aerobic conditions, growth of STM_*modA* was equivalent to that of wild-type PAO1. In contrast, under anaerobic conditions where NO_3_ was provided as the terminal electron acceptor, the STM_*modA* mutant was unable to perform productive growth (Fig. 2a). However, when the growth medium was supplemented with 100 μM of sodium molybdate, mutant growth was restored to wild-type levels. Molybdate supplementation had no effect on PAO1 growth. Wild-type *modABC* was provided in *trans* to the mutant strain for complementation analysis using the expression vector pUCP19. The complemented STM_*modA* strain showed PAO1-like in vitro growth levels under anaerobic conditions (Fig. 2b), which confirmed that the disruption of *modA* is responsible for the STM_*modA* mutant phenotype in anaerobic conditions and that the ModABC transporter is essential for anaerobic growth. To confirm that this anaerobic growth defect was due to the malfunction of one or all nitrate reductases, residual nitrates in the growth medium were quantified (Fig. 2c). In the presence of nitrate reductase activity, nitrate concentration is expected to decrease due to bacterial uptake and subsequent reduction. After overnight growth, residual nitrates in the growth medium of wild-type PAO1 were completely consumed. In contrast, for the STM_*modA* mutant, nitrate concentration at the end of the experiment was identical to the initial concentration, demonstrating that no denitrification had occurred. When supplemented with molybdate, STM_*modA* showed PAO1-like nitrate consumption. Thus, nitrate consumption is molybdate-dependent in the STM_*modA* mutant.

It was previously demonstrated that *mod* mutations in *E.coli* caused pleiotropic effects on molybdo enzyme activity, including nitrate reductase activity [33]. These effects were reversible in the presence of high concentrations of molybdate, which can be internalized by the sulfate transport system under appropriate conditions [16, 34]. However, sulfur compounds have been shown to inhibit the sulfate transport system, thus in protein-rich medium (such as LB), molybdate is likely internalized by another, less specific transporter, presumably the selenite transport system [34]. A novel permease (PerO) internalizing molybdate at micromolar concentrations was identified in *Rhodobacter capsulatus* [35]. PerO also imports sulfate, tungstate and vanadate, suggesting a general oxyanion transporter function. PerO has 30 % AA sequence identity with *P.aeruginosa* PAO1 PA3839, which may be responsible for the nonspecific uptake of molybdate when added at micromolar concentrations, as was done here for the *modA* mutant.

### *modA* and biofilm formation

**Fig. 3:**
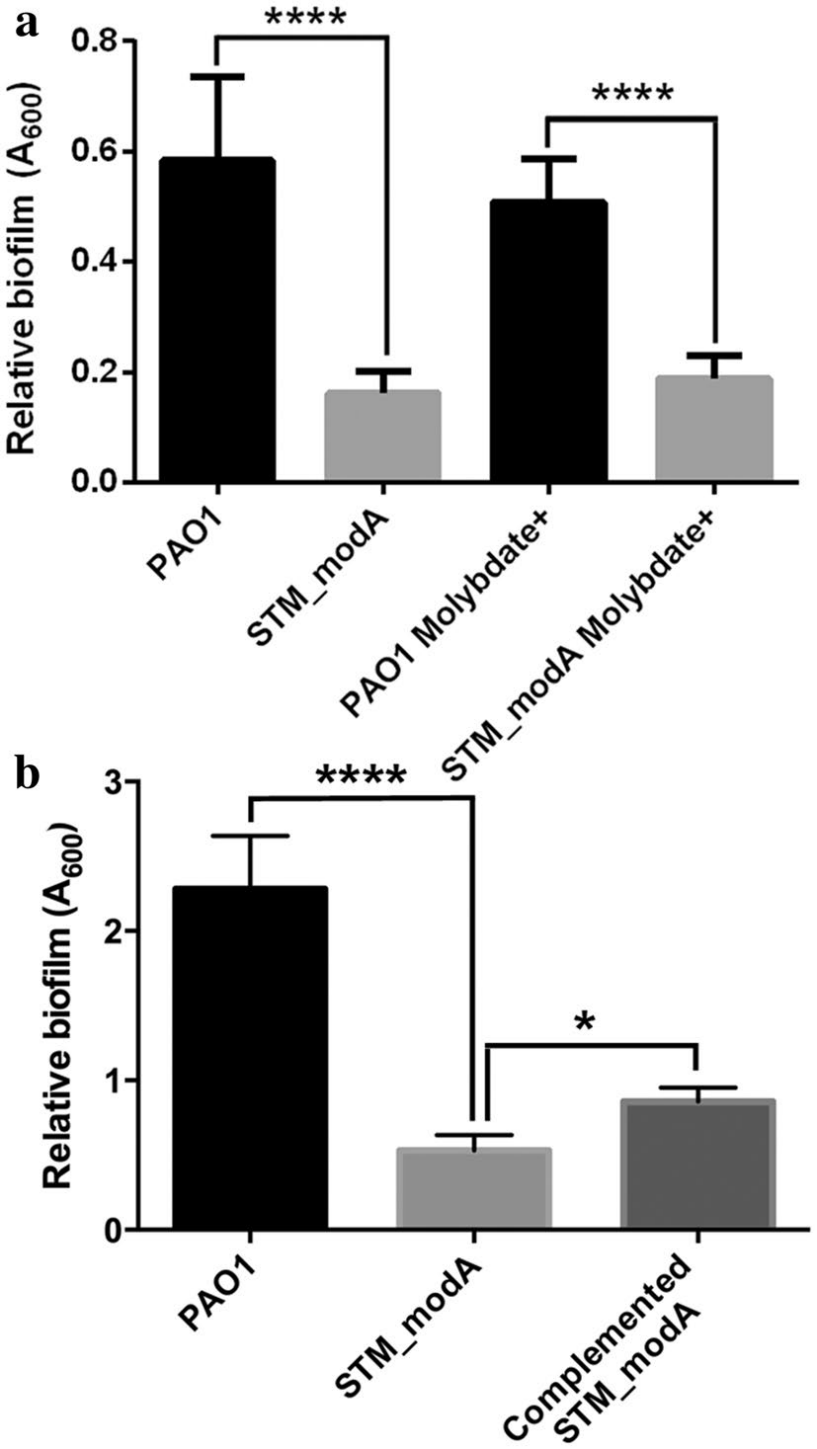
Quantification of biofilm formation. **a** Relative biofilm production for wild-type PAO1 and the STM_*modA* mutant, in M63 medium supplemented with 100 μM sodium molybdate when indicated. Supplementation with KNO_3_ had no impact on these results. Statistically significant values were obtained using an ANOVA and Fisher’s LSD tests in GraphPad Prism 6.0 (****p < 0.0001). Biofilm formation was quantified by measuring the absorbance of crystal violet-stained attached cells, after a 6-hour incubation [25]. The data shown represent 8 replicates per strain for one of three consistent assays. STM_*modA* produces significantly less biofilm than PAO1 and this was not restored by the addition of molybdate and a source of electrons for anaerobic growth (denitrification). **b** Relative biofilm for PAO1, STM_*modA* and the complemented STM_*modA* in M63 medium. Biofilm formation was partly restored in the complemented strain (ANOVA and Fisher’s LSD, *p = 0.01, ****p < 0.0001)

The STM_*modA* mutant strain was tested in vitro for biofilm formation, H_2_O_2_ sensitivity, heat shock, proteolytic, lipolytic and hemolytic activities, swarming, twitching and swimming motilities, as well as pyocyanin and pyoverdine production. Among all tested phenotypic traits, only biofilm formation presented a statistically significant difference between STM_*modA* and wild-type PAO1 (Fig. 3a). To confirm whether the weak biofilm formation observed in STM_*modA* was caused by a defect in anaerobic growth in the presence of trace amounts of molybdate, the M63 medium was supplemented with molybdate alone, and with molybdate + KNO_3_. Results showed that the defect was independent from molybdate supplementation, which implies that reduced biofilm formation in STM_*modA* is not related to its growth defect in anaerobic conditions. When complemented using the recombinant plasmid encoding wild-type ModABC, biofilm formation was only partially restored (Fig. 3b). These results differ from those of Pederick and colleagues [18], according to which biofilm production was unaltered in the *modA* deletion mutant in 5 ml 24 h static cultures. This may be due, at least in part, to important differences between protocols, as biofilm formation is extremely sensitive to growth conditions e.g. [36]. Namely, the significant differences in volume and duration of the assay may have led to different oxygen gradients. If that was the case, it would imply that results from both studies on biofilm formation highlight the complex nature of this process in *P. aeruginosa* and should be interpreted with caution. However, since this study focusses on an insertional mutant, we cannot rule out the possibility of non-specific effects of the mutation, which may also explain this discrepancy. For instance, the gene adjacent to *modA* in the genome of strain PAO1, which was annotated as a putative transcriptional regulator, may be affected by the transposon insertion and result in pleiotropic effects. Further investigation would be required to confirm this gene’s function. It is noteworthy, however, that partial phenotype and/or virulence rescue was previously observed in other complemented *P. aeruginosa* mutants see reference [37], namely in mouse models of acute pneumonia and burn sepsis using the same plasmid [38]. Therefore, it is possible that a more general mechanism, which remains to be described, is responsible for these observations.

### *modA* is important for virulence during chronic lung infection

**Fig. 4:**
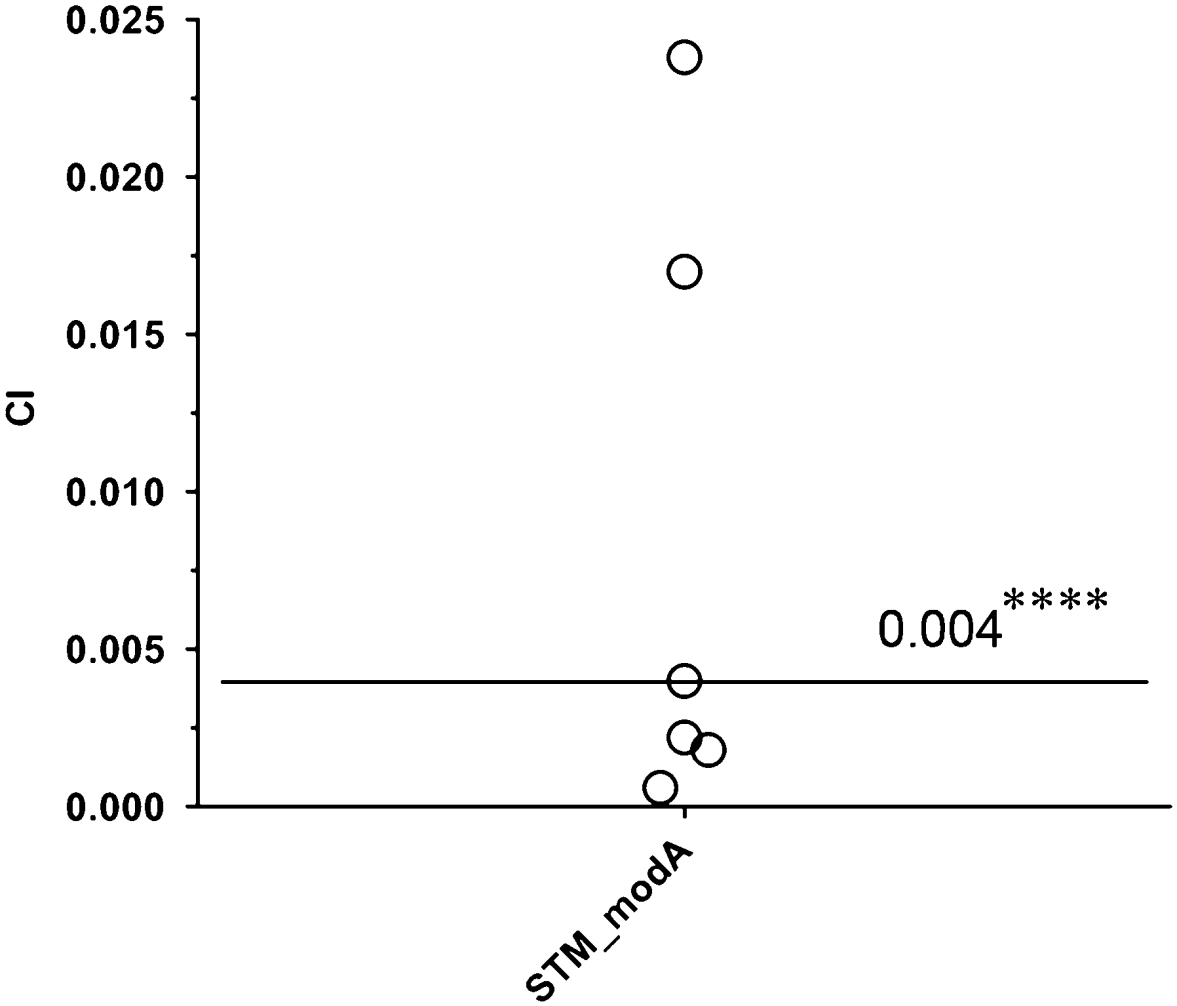
In vivo competitive index (CI) of *P. aeruginosa* STM_*modA* in the rat lung. CI of STM_*modA* against wild-type PAO1 was determined 7 days post-infection. Each *circle* represents the CI for a single animal. A CI of less than *one* indicates a virulence defect. The geometric mean of the CI for all rats is shown as a *solid* line (****p < 0.0001, Mann–Whitney sum test)

In the original screening experiment where the STM_*modA* mutant was targeted as defective for in vivo maintenance, pools of 72 STM mutant strains were tested simultaneously [15]. Therefore, further investigation was required to precisely describe the involvement of *modA* in the virulence of *P.aeruginosa* PAO1. An in vivo competitive index (CI) in the rat model of chronic lung infection was performed using both wild-type strain PAO1 and the STM_*modA* mutant. As depicted in Fig. 4, at 7 days post-infection, we recorded an in vivo CI value of 0.004, which represents a 250-fold decrease in mutant colony forming units (CFU) compared to the wild-type strain. Thus, STM_*modA* is unable to maintain a chronic lung infection in the rat model when in competition with wild-type PAO1. However, in aerobic combined culture conditions, PAO1 does not outcompete STM_*modA*, with an in vitro CI value of ~1.0 after 22 h. In addition, STM_*modA* was capable of normal growth in minimal M9 medium; hence it is not an auxotroph mutant. This contrast between in vitro and in vivo proliferation of the STM_*modA* mutant strain clearly stresses that mutation of *modA* has consequences that are relatively specific to the context of infection and probably influenced by the interaction with the host.

### *modA* is important for virulence in the amoeba model

In order to further investigate the idea that the role of *modA* in virulence may be affected by interaction with the host, we decided to use a markedly different host model. *D.discoideum* is an alternative model for the study of bacterial virulence where amoebae feed by bacterial uptake using phagocytosis [28, 39]. *P.aeruginosa* has universal virulence factors used to infect phylogenetically diverse hosts, which makes the amoeba model particularly well suited to study its virulence [28, 39]. Previously identified virulence factors using this model, related to quorum sensing and the type III secretion system, were also essential for infection in mammalian and insect models [40]. Many bacterial virulence factors are active against predation by *D. discoideum*, thus virulence is assumed to be inversely proportional to amoeba grazing [29, 39]. In addition, the predation assay is performed in aerobic conditions where no growth difference has been noticed between PAO1 and the STM_*modA* mutant.

**Fig. 5:**
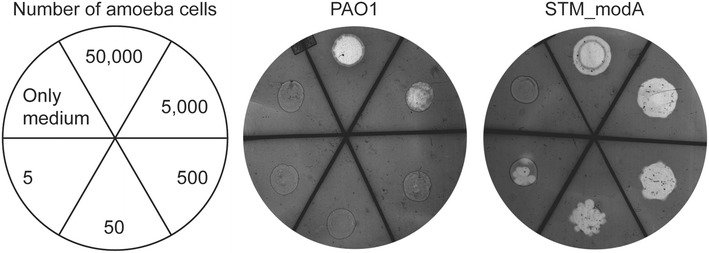
*D. discoideum* predation assay. STM_*modA* exhibits a loss of resistance to amoeba predation when compared to the wild-type PAO1. Numbers represent *D. discoideum* cells deposited on the bacterial lawn. The number of phagocytic plaques is inversely proportional to predation resistance. PAO1 was grazed by a minimum of 5000 cells while STM_*modA* was sensitive to predation by only five cells. Results were consistent among three independently performed assays

As shown in Fig. 5, the STM_*modA* mutant was very susceptible to amoeba predation as shown by the presence of phagocytic plaques with only five *D. dictostelium* cells on a bacterial lawn of this mutant compared to PAO1, where a minimum of 5000 *D. dictostelium* cells was required to observe a phagocytic plaque. These results confirmed the virulence defect of the STM_*modA* mutant. As *D. discoideum* is an acute infection model while agar-embedded bacteria in the rat lung represent a chronic lung infection model, these two assays highlight the fact that *modA* may be important for the global virulence network of *P. aeruginosa*, even in aerobic conditions.

### Metabolic and molecular basis of STM_*modA* multi-host virulence

Results presented here independently confirmed that transport of the trace anion molybdate is essential for the activity of molybdoenzymes such as nitrate reductase in *P. aeruginosa* [18]. In anaerobic conditions, molybdate uptake was restored in STM_*modA* after the addition of molybdate to the culture medium, which suggests less specific internalization of the molecule by a system other than ModABC. The lack of anaerobic growth in STM_*modA* was hypothesized to be directly linked to the maintenance defect observed in the rat model of chronic lung infection (CI = 0.004) because this model mimics CF lung infections, which present evidence of oxygen-depletion [7–9, 41, 42]. This hypothesis, however, does not hold in the case of the amoeba predation assay, where a strong virulence defect was observed in at least partly aerobic conditions; oxygen availability may be limited in a fully grown bacterial lawn. For the biofilm formation assay, our results showed that STM_*modA* is unable to produce as much biofilm as PAO1 in vitro, and that this effect is molybdate-independent. As biofilm is a crucial component of *P.aeruginosa* chronic lung infections [43], reduced biofilm production in STM_*modA* may be related to the virulence defect observed in the rat model of chronic lung infection, in conjunction with anaerobic growth defectiveness. The virulence defect observed in the amoeba model, on the other hand, could be linked to this biofilm defect alone, as for other biofilm-defective mutants shown to have attenuated virulence in the *D. dictostelium* model [44]. Even without consideration for the biofilm results, which have been discussed at length earlier, the amoeba predation assay suggests that the impact of the mutation in *modA* may go beyond its effect on anaerobic growth, perhaps due to pleiotropic effects of this gene.

This study is not the first report of an impact on virulence for a gene implicated in molybdate homeostasis in *P.aeruginosa*. PA1006 encodes a protein of unknown function implicated in molybdate homeostasis that is critical for biofilm maturation and virulence in a lettuce model and in a burned mouse acute virulence model [45]. Other genes implicated in molybdenum utilization and transport (several *moe*, *moa* and *mod* genes) have been linked to a pathogenesis defect in *Mycobacterium tuberculosis* [46]. These genes were required for intracellular growth in macrophages or maintenance in the organs. Interestingly, *modA* has also been identified in a STM experiment with *M. tuberculosis* using a mouse model of acute lung infection with an intravenous administration route [47].

## Conclusions

We have shown that the inactivation of the *modA* gene in a transposon mutant caused a significant defect in *P. aeruginosa* PAO1 for the establishment of chronic lung infection and for resistance to *D.dictostelium* predation. This study complements previous work [18] in providing evidence that molybdate uptake is important for anaerobic growth and multi-host virulence in *P. aeruginosa*. Since the capacity to thrive in anaerobic conditions is relevant to CF lung infections, the *modABC*-encoded transporter may represent a potential target for therapeutic intervention and deserves further investigation.

## Additional file

10.1186/s13104-016-1840-x Primers used for sequencing of the insertion site, complementation and sequencing of the vector for complementation.
