# Supplementary material for: Molybdate transporter ModABC is important for Pseudomonas aeruginosa chronic lung infection
Source: BMC Res Notes. 2016 Jan 12;9:23. doi: 10.1186/s13104-016-1840-x (PMC4710039; doi:10.1186/s13104-016-1840-x)
Supplement: Supplementary file 1 — 10.1186/s13104-016-1840-x Primers used for sequencing of the insertion site, complementation and sequencing of the vector for complementation. [file 13104_2016_1840_MOESM1_ESM.pdf]

Table S1. Primers used for sequencing of the insertion site, complementation and sequencing of the vector for complementation.

**Primer**	**Relevant sequence (5’-3’)**	**Use**
PA1863_R	CGT ACT GTC GAG TGG CAA C	Sequencing of tn*5* insertion site
putKana2	CGTACTGTCGAGTGGCAACG	(Same as above)
pUCP19-1	AGT GAA TTC GAC CTC GGT ACG GCA TCC GGC CCT GCG TTT C	Complementation: cloning of *mod*ABC into pUCP19 using Gibson assembly
pUCP19-2	GCC AAG CTT GCA TGC CTG CAG AGA TCA TGC GCG GCG CG	(Same as above)
modABC-1	GCG AGA AAC CGC TGG AGG CTG CCG CGA C	(Same as above)
modABC-2	AGC CTC CAG CGG TTT CTC GCC GAT CGC C	(Same as above)
Insert1	AGG GTC AGC AGG CCG TAG	Complementation: double-strand sequencing of the *mod*ABC insert into pUCP19
Insert1-rev	CTA CGG CCT GCT GAC CCT	(Same as above)
Insert2	GAT ACA GCG CAG GGC A AGT G	(Same as above)
Insert2-rev	CAC TTG CCT GCG CTG TAT C	(Same as above)
Insert3	AGC AGG TAG AAG CCG ATC AC	(Same as above)
Insert3-rev	GTG ATC GGC TTC TAC CTG CT	(Same as above)
Insert4	AGA GGA ACA CCT GGA ACG G	(Same as above)
Insert4-rev	CCG TTC CAG GTG TTC CTC T	(Same as above)
